# Increased incidence of glucose disorders during pregnancy is not explained by pre-pregnancy obesity in London, Canada

**DOI:** 10.1186/1471-2393-10-85

**Published:** 2010-12-24

**Authors:** Margie H Davenport, M Karen Campbell, Michelle F Mottola

**Affiliations:** 1 R. Samuel McLaughlin Foundation Exercise and Pregnancy Lab, School of Kinesiology, The University of Western Ontario, London, Ontario, N6A 3K7, Canada; 2 Department of Epidemiology and Biostatistics, The University of Western Ontario, London, Ontario, N6A 3K7, Canada; 3 Department of Obstetrics and Gynecology, The University of Western Ontario, London, Ontario, N6A 3K7, Canada; 4 Department of Paediatrics, The University of Western Ontario, London, Ontario, N6A 3K7, Canada; 5 Childrens Health Research Institute, The University of Western Ontario, London, Ontario, N6A 3K7, Canada; 6 Department of Anatomy and Cell Biology, The University of Western Ontario, London, Ontario, N6A 3K7, Canada

## Abstract

**Background:**

The increasing incidence of impaired glucose tolerance (IGT), gestational diabetes (GDM) and type 2 diabetes (T2D) during pregnancy was hypothesized to be associated with increases in pre-pregnancy body mass index (BMI). The aims were to 1) determine the prevalence of IGT/GDM/T2 D over a 10 year period; 2) examine the relationship between maternal overweight/obesity and IGT/GDM/T2D; and 3) examine the extent to which maternal metabolic complications impact maternal and fetal pregnancy outcomes.

**Methods:**

Data arose from a perinatal database which contains maternal characteristics and perinatal outcome for all singleton infants born in London, Canada between January 1, 2000 and December 31, 2009. Univariable and multivariable odds ratios (OR) were estimated using logistic regression with IGT/GDM/T2 D being the outcome of interest.

**Results:**

A total of 36,597 women were included in the analyses. Population incidence of IGT, GDM and T2 D rose from 0.7%, 2.9% and 0.5% in 2000 to 1.2%, 4.2% and 0.9% in 2009. The univariable OR for IGT, GDM and T2 D were 1.65, 1.52 and 2.06, respectively, over the ten year period. After controlling for maternal age, parity and pre-pregnancy BMI the OR did not decrease. Although there was a positive relationship between pre-pregnancy BMI and prevalence of IGT/GDM/T2 D, this did not explain the time trends in the latter. Diagnosis of IGT/GDM/T2 D increased the risk of having an Apgar score <7 at 5 minutes, which was partially explained by gestational hypertension, high placental ratio, gestational age and large for gestational age babies.

**Conclusions:**

We found a significant increase in the incidence of IGT/GDM/T2 D for the decade between 2000-2009 which was not explained by rising prevalence of maternal overweight/obesity.

## Background

Maternal glucose disorders (MGD) during pregnancy include gestational diabetes mellitus (GDM), impaired glucose intolerance (IGT) and pre-pregnancy type 2 diabetes. GDM is defined as glucose intolerance with onset or first diagnosis during pregnancy [1,2]. This is a common disease affecting 3-4% of pregnancies in Canada [1]. Recent data from the United States and Australia have indicated that the prevalence of GDM is on the rise. However, the underlying cause is unknown [3-12]. Postulated mechanisms include the concomitant rise in obesity [12,13] and decrease in physical activity [14,15]. In 2000, 30% of American adults were obese (body mass index (BMI) ≥30 kg/m^2^) representing a 23% increase over the previous decade [16]. In a study of nine US states, the prevalence of maternal obesity increased 69.3% from 1993-2003 [17]. Several studies have demonstrated that MGD is strongly related to maternal obesity [18,19]. However, none of the studies previously examining the time trend of MGD had pre-pregnancy BMI available in their database [3-11]. Therefore, ours is the first to examine the relationship between pre-pregnancy BMI and changes in the prevalence of MGD.

The objectives of the present study were: 1) to describe the annual prevalence of MGD (including GDM, type 2 diabetes and IGT) over a 10 year period; 2) to examine the relationship between annual prevalence of maternal overweight/obesity and MDG prevalence to determine the extent to which they are associated; and 3) to examine, at the individual level, the extent to which maternal metabolic complications impact on maternal and fetal pregnancy outcomes. We hypothesized that, between 2000-2009, the prevalence of maternal glucose disorders will have increased, especially in women with a BMI ≥25 kg/m^2^. It was also hypothesized that the time trend of maternal glucose disorders would be explained by the time trend of overweight/obesity. Finally, we hypothesized that the offspring of pregnancies affected by maternal glucose disorders would have an increased incidence of pregnancy complications compared with the normal obstetrical population.

## Methods

The following protocol has been approved by the Research Ethics Board for the Review of Health Sciences Research Involving Human Subjects at the University of Western Ontario. We conducted a retrospective cohort study using data from the Saint Joseph's Health Care Perinatal Database which contains data from medical records, including delivery and neonatal information. Data from all births occurring in London, Canada are prospectively collected and entered into this large perinatal database by a dedicated research assistant. The study population was selected based on the following criteria: delivery between January 1, 2000 and December 31, 2009 (n = 36,597), singleton pregnancy and residence in London, Canada. Women diagnosed with type 1 diabetes prior to pregnancy were removed from the dataset. The following data were used for analyses: maternal characteristics (age, parity, height (measured during prenatal visit), pre-pregnancy weight (from maternal recall), excessive weight gain, previous GDM or pre-existing type 2 diabetes, pre-existing hypertension, gestational hypertension (including pregnancy induced hypertension and pre-eclampsia), IGT and diagnosis of GDM - which may include previously undiagnosed type 2 diabetes), infant characteristics (gestational age, birth and placental weight) and pregnancy outcomes (type of delivery and Apgar scores). After the data were checked for errors, all identification (including names and hospital record numbers) was stripped to ensure anonymity of the data. Large for gestational age (LGA) and small for gestational age (SGA) were identified using Canadian birth weight for gestation distributions [20]. A ratio dividing placental weight (g) by infant birth weight (g) was determined. Maternal pre-pregnancy weight and height were taken from medical records. Maternal pre-pregnancy BMI was calculated using pre-pregnancy weight (kg)/[height (m)]^2^. Excessive pregnancy weight gain was defined as weight gain from pre-pregnancy weight to delivery weight ≥20 kg in the database. Type 2 diabetes, GDM and IGT were taken directly from diagnosis entered into the database. All pregnant women in Canada are screened and diagnosed for these diseases according to guidelines set by the Canadian Diabetes Association [1,2].

All analyses were performed using the Statistical Analysis System 9.1.3 (SAS Institute, Cary, NC) and Statistical Package for the Social Sciences, version 16 (SPSS Inc, Chicago, Ill). The annual prevalence of maternal glucose disorders from 2000-2009 (Objective 1) within strata (by year, body mass index (BMI), age and/or parity) was estimated as a proportion and standard error of the estimate. To address Objective 2, prevalence of maternal glucose disorders by pre-pregnancy BMI categories (underweight (< 18.5 kg/m^2^), normal weight (18.5-24.9 kg/m^2^), overweight (25-29.9 kg/m^2^) and Class I, II and III obese (30-34.9 kg/m^2^, 35-39.9 kg/m^2 ^and ≥40 kg/m^2^, respectively) were summarized using frequency distributions. Repeated measures ANOVA was used to determine the increase in overweight/obesity over time. P < 0.05 indicates statistical significance. Univariable and multivariable odds ratios were estimated using logistic regression with the outcome of interest being MGD (IGT, GDM and type 2 diabetes). Predictive factors were entered in a blockwise fashion in order to allow for a careful examination of the relative contributions of each predictive factor to the relationship between year and MGDs. Year, maternal age and parity were entered in the first block, BMI in the second block and excessive weight gain in the third block. To address objective 3, frequency distributions and mean ± SD of 1) IGT; 2) GDM; 3) type 2 diabetes; and 3) no metabolic disorder were created. Univariable and multivariable odds ratios were estimated using logistic regression with the outcome of interest being Apgar <7 at 5 minutes after birth. Apgar <7 at 5 minutes after birth was chosen as a proxy for neonatal well-being, because a variety of clinical events that affect the well-being of the infant will be reflected in the Apgar score [21].

## Results

We examined data from 36,597 women who gave birth between Jan 1, 2000 and Dec 31, 2009 in London, Ontario, Canada. Description of the sample follows with mean ± standard deviation (min-max). The women were 29.5 ± 5.5 (14.1-47.9) years old, 165 ± 10 (103-195) cm tall, with a pre-pregnancy body mass of 67.0 ± 15.9 (31.8 - 189.6) kg and a pre-pregnancy BMI of 24.7 ± 5.5 (14.6-67.5) kg/m^2^. Pre-pregnancy BMI was unavailable for 8,611 women. These women were not included in the analysis.

### Incidence of maternal glucose disorders from 2000-2009

Pregnancies affected by type 2 diabetes increased significantly from 0.5% in 2000 to 0.9% in 2009 (p = 0.001). The prevalence of GDM significantly increased over the same period from 2.9% to 4.2% (p = 0.002) and the prevalence of IGT increased from 0.7% to 1.2% (p = 0.001).

The prevalence of pre-pregnancy overweight and obesity did not significantly change between 2000 and 2009 and were, respectively, 22.1% and 14.4% in 2000 and 22.2% and 16.0% in 2009. The p-values attached to changes in prevalence were p = 0.79 for overweight and p = 0.43 for obesity. Thus, there was not an association with the time trend in MGT. However, ignoring time trends, there was a positive relationship between BMI and maternal glucose disorders in that as BMI category increased, the prevalence of maternal glucose disorders also increased (Table 1).

**Table 1:** Body mass index and metabolic disorders.

	< 18.5 (N = 1618)	18.5-24.9 (N = 15898)	25.0-29.9 (N = 6272)	30.0 - 34.9 (N = 2571)	35.0 - 39.9 (N = 1067)	> 40.0 (N = 560)
Previous GDM	9 (0.6%)	132 (0.8%)	129 (2.1%)	81 (3.2%)	58 (5.4%)	39 (7.0%)

Type 2 Diabetes	5 (0.2%)	52 (0.3%)	48 (0.8%)	28 (1.1%)	23 (2.2%)	23 (4.1%)

IGT	21 (1.3%)	110 (0.7%)	87 (1.4%)	37 (1.4%)	17 (1.6%)	11 (2.0%)

GDM	5 (0.3%)	316 (2.0%)	299 (4.8%)	193 (7.5%)	111(10.4%)	95 (17.0%)

Values are number diagnosed (%). GDM, gestational diabetes mellitus; IGT, impaired glucose tolerance.

Univariable and multivariable associations of year, maternal age, parity, BMI and excessive weight gain with MGDs are presented in Table 2. Univariable analyses indicate a 65%, 52% and 206% increase in the prevalence of IGT (OR: 1.65, 95% CI: 1.12 - 2.43), GDM (OR: 1.52, 95% CI: 1.25-1.83)) and type 2 diabetes (OR: 2.06, 95%CI: 1.29-3.27), respectively, for the decade between 2000 and 2009 with a slight amplification of these ORs after control for covariates. Therefore, the increase in the prevalence of maternal glucose disorders over time was not explained by BMI nor was it explained by excessive pregnancy weight gain (IGT aOR: 2.09, 95% CI: 1.36-3.20; GDM aOR: 1.68, 95% CI: 1.35-2.10; type 2 diabetes aOR 2.33, 95% CI: 1.38-3.92) (Table 2). Strong univariable and multivariable associations were demonstrated between BMI and MGDs.

**Table 2:** Univariable and multivariable analysis of factors associated with impaired glucose tolerance (IGT), gestational diabetes mellitus (GDM) and type 2 diabetes.

		Univariable	Multivariable	Multivariable + BMI	Multivariable + BMI + Excessive Weight Gain
Year	IGT	1.65 (1.12-2.43)	1.65 (1.12-2.43)	2.09 (1.36-3.20)	2.09 (1.36-3.20)
	GDM	1.52 (1.25-1.83)	1.52 (1.12-2.43)	1.68 (1.35-2.10)	1.68 (1.35-2.10)
	Type 2	2.06 (1.29-3.27)	2.07 (1.30-3.28)	2.33 (1.38-3.92)	2.34 (1.39-3.94)

Maternal Age (≥35 vs. <35)	IGT	1.50 (1.13-1.96)	1.60 (1.22-2.10)	1.40 (1.03-1.9)	1.40 (1.03-1.9)
	GDM	2.46 (2.18-2.77)	2.39 (2.12-2.70)	2.31 (2.01-2.66)	2.31 (2.0-2.65)
	Type 2	2.11 (1.58-2.83)	2.07 (1.54-2.79)	1.97 (1.41-2.76)	1.99 (1.42-2.79)

Parity (≥ 1 vs. 0)	IGT	0.82 (0.66-1.03)	0.77 (0.61-0.96)	0.75 (0.58-0.96)	0.75 (0.58-0.96)
	GDM	1.31 (1.17-1.46)	1.13 (1.01-1.27)	0.95 (0.83-1.08)	0.94 (0.83-1.08)
	Type 2	1.23 (0.94-1.61)	1.10 (0.84-1.44)	0.91 (0.67-1.23)	0.93 (0.69-1.26)

BMI (25.0-29.9 vs. <25.0)	IGT	2.22 (1.69-2.94)		2.24 (1.69-2.92)	2.24 (1.69-2.96)
	GDM	2.58 (2.21-3.03)		2.52 (2.15-2.96)	2.52 (2.15-2.96)
	Type 2	2.54 (1.67-3.61)		2.41 (1.64-3.55)	2.41 (1.64-3.55)

BMI: (≥30.0 vs. <25.0)	IGT	2.65 (1.95-3.6)		2.68 (1.97-3.65)	2.68 (1.97-3.65)
	GDM	5.50 (4.74-6.39)		5.43 (4.67-6.31)	5.39 (4.63-6.27)
	Type 2	6.03 (4.26-8.54)		5.96 (4.21-8.44)	6.11 (4.30-8.67)

Excessive Weight Gain (> 20 kg)	IGT	1.06 (0.75-1.48)			1.00 (0.70-1.42)
	GDM	0.77 (0.64-0.92)			0.91 (0.74-1.11)
	Type 2	1.16 (0.79-1.71)			1.36 (0.90-2.05)

Multivariable analysis includes year, maternal age and parity.

Multivariable + BMI analysis includes year, maternal age, parity and BMI.

Predictive factors were entered in a blockwise fashion in order to allow for a careful examination of the relative contributions of each predictive factor to the relationship between year and MGDs. Year, maternal age and parity were entered in the first block, BMI in the second block and excessive weight gain in the third block.

### Maternal glucose disorders and pregnancy outcome

Diagnosis of IGT or GDM during pregnancy was associated with an increased incidence of of LGA, caesarean section delivery and gestational hypertension (Table 3) whereas diagnosis of type 2 diabetes during pregnancy was associated with increased frequency of LGA, Apgar <7 at 5 minutes, caesarean section delivery and gestational hypertension. The incidence of SGA is slightly lower. Univariable analysis indicates that the risk of having an Apgar <7 at 5 minutes after delivery is higher in IGT (OR 1.87, 95% CI: 1.36 - 2.56), GDM (OR 1.50, 95% CI: 1.27 - 1.77), type 2 diabetes (OR 2.99, 95% CI: 2.17 - 4.12), BMI ≥30 kg/m^2 ^(OR 1.54, 95% CI: 1.38 - 1.71), high placental ratio (OR 1.93, 95% CI: 1.76 - 2.12) and gestational hypertension (OR 1.69, 95% CI: 1.51 - 1.89) (Table 4). After controlling for maternal age, BMI, parity, type 2 diabetes, GDM and excessive weight gain, multivariable analysis of factors associated with Apgar at 5 minutes <7 were unchanged (Table 4). Further multivariable analysis of factors associated with Apgar at 5 minutes <7 indicated that the increased risk of IGT, GDM and type 2 diabetes diagnosis is partially explained by gestational hypertension, high placental ratio, gestational age and LGA (Table 4, multivariable model 3).

**Table 3:** The impact of maternal glucose disorders on maternal and fetal pregnancy outcomes.

	IGT	GDM	T2D	Normal
	
	Percent or Mean	Percent or Mean	Percent or Mean	Percent or Mean
	N = 317	N = 1342	N = 224	N = 34714
Gestational Age (weeks)	38.7 ± 1.8	38.2 ± 1.9	37.1 ± 2.4	39.0 ± 2.0

Birth Weight (g)	3462 ± 568	3442 ± 637	3533 ± 806	3415 ± 577

Placental Weight (g)	699 ± 149	714 ± 200	736 ± 206	674 ± 171

Placental Ratio	0.2095 ± 0.0340	0.2095 ± 0.0411	0.2132 ± 0.0475	0.1993 ± 0.0421

LGA	18.6%	21.8%	43.8%	11.2%

SGA	6.6%	7.2%	2.7%	8.4%

Apgar 5 Minutes <7	2.5%	2.5%	3.1%	1.6%

C/S Delivery	31.2%	35.8%	57.1%	20.2%

gestational hypertension	15.5%	15.6%	28.1%	7.5%

Values are mean ± SD or number diagnosed (%). GDM, gestational diabetes mellitus; T2 D, type 2 diabetes; LGA, large for gestational age; SGA, small for gestational age; Apgar, appearance, grimace, pulse, activity, respiration; C/S, caesarean.

**Table 4:** Univariable and multivariable analysis of factors associated with Apgar scores less than 7 at 5 minutes.

	Univariable	Multivariable	Multivariable	Multivariable
		Model 1	Model 2	Model 3
Type 2 Diabetes	2.99 (2.17-4.12)	2.41 (1.66-3.48)	2.40 (1.66-3.48)	1.29 (0.85-1.95)

Maternal Age (≥35 vs. <35)	1.01 (1.00 - 1.02)	1.02 (1.01-1.03)	1.02 (1.01-1.03)	1.02 (1.01-1.03)

BMI (25.0-29.9 vs. <25.0)	1.22 (1.11 - 1.35)	1.22 (1.10-1.35)	1.22 (1.10-1.35)	1.25 (1.12-1.41)

BMI: (≥30.0 vs. <25.0)	1.54 (1.38 - 1.71)	1.53 (1.36-1.71)	1.53 (1.37-1.71)	1.43 (1.25-1.63)

Parity (≥ 1 vs. 0)	0.61 (0.57 - 0.66)	0.55 (0.50-0.60)	0.55 (0.51-0.60)	0.55 (0.50-0.61)

IGT	1.87 (1.36-2.56)	1.81 (1.30-2.53)	1.81 (1.30-2.53)	1.49 (1.03-2.15)

GDM	1.50 (1.27 - 1.77)	1.25 (1.03-1.52)	1.25 (1.03-1.52)	0.95 (0.76-1.20)

Excessive Weight Gain	1.08 (0.97 - 1.21)		1.03 (0.92-1.16)	1.10 (0.85-1.95)

Gestational Hypertension	1.69 (1.51 - 1.89)			0.99 (0.85-1.16)

High Placental Ratio	1.93 (1.76 - 2.12)			1.37 (1.23-1.539)

Gestational Age	0.79 (0.78 - 0.80)			0.82 (0.81-0.84)

LGA	1.44 (1.30 - 1.59)			1.55 (1.35-1.77)

Model 1 includes maternal age, body mass index (BMI), and parity.

Model 2 includes Model 1 plus impaired glucose tolerance (IGT), gestational diabetes mellitus (GDM), type 2 diabetes and excessive weight gain.

Model 3 includes Model 2 plus gestational hypertension, placental ratio, gestational age and large for gestational age (LGA).

## Discussion

The results indicated that in London, Canada the prevalence of maternal glucose disorders increased by 65% for IGT, 52% for GDM and 206% for type 2 diabetes between 2000 and 2009. While maternal glucose disorders were shown to be associated with overweight and obesity, the temporal increase in maternal glucose disorders over the ten year period was not explained by similar increases in BMI. Examination of outcomes indicated that being diagnosed with IGT, GDM and type 2 diabetes did increase the risk of having an Apgar score <7 at 5 minutes.

To our knowledge, this is the first study to analytically examine the relationship between prevalence of maternal glucose disorders and obesity over time. Nine studies previously examined the changing prevalence in GDM during various time points between 1979 and 2005 [3-12]. One study found that the prevalence of GDM was stable between 1999 - 2005 in a Southern California population while the prevalence of type 2 diabetes increased [11]. The remaining studies demonstrated an increase in GDM that ranged from 12% [5] to 127% [4]. While each of these studies was able to examine the influence of ethnicity on the rising prevalence of GDM, most found that this increase was independent of ethnicity or only partially explained by ethnicity. None of the previous studies examining the time trend of maternal glucose disorders had access to pre-pregnancy BMI to determine if overweight/obesity played a role. As predicted by the literature, we found that elevated BMI was a major risk factor for developing GDM [22]. However, we also found that BMI ≥25 kg/m^2 ^did not increase significantly between 2000 and 2009 in our obstetrical population. These results are surprising as they are in contrast to Kim *et al *[17] who examined obesity rates in nine US states and found that the prevalence of maternal obesity (BMI >29 kg/m^2^) and overweight (BMI 26.0 - 29.0 kg/m^2^) increased by 69.3% and 18.6% between 1993 and 2003, respectively. In the present study, after controlling for maternal age, parity and pre-pregnancy BMI, the impact of year was a 209% increase in IGT, a 168% increase in GDM and a 233% increase in pre-existing type 2 diabetes. These results suggest that there are other important variables affecting the prevalence of maternal glucose disorders. Postulated mechanisms include physical inactivity or excessive intake of dietary fat [23,24]. Further research is required.

The results of the univariable analyses in the present study found that the risk of low Apgar scores was related to diagnosis of IGT, GDM, T2 D, obesity, gestational hypertension and high placental ratio. However, after controlling for confounding factors, the low Apgar scores associated with IGT, GDM and T2 D were partially explained by concurrent diagnosis of gestational hypertension, having a high placental ratio, gestational age and delivering an LGA infant. It was further found that pre-pregnancy obesity was an independent risk factor for low Apgar scores. Therefore, it is important that clinicians consider maternal obesity alone as an independent risk factor for adverse pregnancy outcomes. Overall, it is important to note that associations with Apgar <7 were modest (OR < 2.0) for all factors, even those with high statistical significance, indicating weak evidence of true associations.

There are three limitations to this paper that should be addressed in future investigations. First, the database does not identify the ethnicity of the women in the sample. According to available 1996 and 2006 Canadian Census [25,26], the ethnic distribution of London has changed modestly between those two years. Previous studies have demonstrated that ethnicity did have a small impact on prevalence of MDG, however, did not fully explain time trends in MDG [3-11]. Since we were not able to assess whether control for ethnicity might modify the estimated impact of other factors in our analyses, the influence of ethnicity on our findings cannot be completely ruled out.

The second limitation of the study was that the database did not indicate if all women in our cohort were screened for GDM. In Canada, it is recommended that all women be screened between 24-28 weeks gestation based on national guidelines by the Canadian Diabetes Association [1,2]. However, the main goal of the present study was to examine the relationship between changing prevalence of maternal glucose disorders and obesity. Therefore, we feel that this is a significant step forward in our understanding of why maternal glucose disorders are increasing in the obstetrical population.

Finally, height and weight data contributing to the calculation came from variable sources, including self-report, therefore misreporting will have influenced the accuracy of BMI. We speculate it may have produced an underestimate in BMI. However, this underestimate is likely to be non-differential in regards to year, and therefore this would not explain the negative finding.

## Conclusion

This is the first study to investigate the relationship between increasing prevalence of maternal glucose disorders and obesity. Contrary to our hypothesis, the results of the present study indicated that the rising prevalence of maternal glucose disorders is not related to increasing BMI.

## Competing interests

The authors declare that they have no competing interests.

## Authors' contributions

MHD contributed to the design, analysis and interpretation of the data and the drafting of the manuscript. MKC and MFM contributed to the design and interpretation of the data and reviewed the manuscript. All authors have read and approved the final manuscript.

## Pre-publication history

The pre-publication history for this paper can be accessed here:

http://www.biomedcentral.com/1471-2393/10/85/prepub

## References

[B1] Canadian Diabetes Association Clinical Practice Guidelines Expert Committee Gestational diabetes mellitus Canadian Medical Association Journal 2003 169 S99 S105

[B2] Canadian Diabetes Association Canadian Diabetes Association 2008 Clinical Practicie Guidelines for the Prevention and Management of Diabetes in Canada Canadian Journal of Diabetes 2008 32 S0 S201

[B3] Ferrara A Kahn HS Quesenberry CP Riley C Hedderson MM An increase in the incidence of gestational diabetes mellitus: Northern California, 1991-2000 Obstet Gynecol 2004 103 526 533 10.1097/01.AOG.0000113623.18286.20 14990417

[B4] Beischer NA Oats JN Henry OA Sheedy MT Walstab JE Incidence and severity of gestational diabetes mellitus according to country of birth in women living in Australia Diabetes 1991 40 Suppl 2 35 38 1748263 10.2337/diab.40.2.s35

[B5] Ishak M Petocz P Gestational diabetes among Aboriginal Australians: prevalence, time trend, and comparisons with non-Aboriginal Australians Ethn Dis 2003 13 55 60 12723013

[B6] Dabelea D Snell-Bergeon JK Hartsfield CL Bischoff KJ Hamman RF McDuffie RS Increasing prevalence of gestational diabetes mellitus (GDM) over time and by birth cohort: Kaiser Permanente of Colorado GDM Screening Program Diabetes Care 2005 28 579 584 10.2337/diacare.28.3.579 15735191

[B7] Moum KR Holzman GS Harwell TS Parsons SL Adams SD Oser CS Spence MR Helgerson SD Gohdes D Increasing rate of diabetes in pregnancy among American Indian and white mothers in Montana and North Dakota, 1989-2000 Matern Child Health J 2004 8 71 76 10.1023/B:MACI.0000025729.65328.73 15198174

[B8] Thorpe LE Berger D Ellis JA Bettegowda VR Brown G Matte T Bassett M Frieden TR Trends and racial/ethnic disparities in gestational diabetes among pregnant women in New York City, 1990-2001 Am J Public Health 2005 95 1536 1539 10.2105/AJPH.2005.066100 16051928 PMC1449393

[B9] Anna V van der Ploeg HP Cheung NW Huxley RR Bauman AE Sociodemographic correlates of the increasing trend in prevalence of gestational diabetes mellitus in a large population of women between 1995 and 2005 Diabetes Care 2008 31 2288 2293 10.2337/dc08-1038 18809630 PMC2584183

[B10] Shand AW Bell JC McElduff A Morris J Roberts CL Outcomes of pregnancies in women with pre-gestational diabetes mellitus and gestational diabetes mellitus; a population-based study in New South Wales, Australia, 1998-2002 Diabet Med 2008 25 708 715 10.1111/j.1464-5491.2008.02431.x 18544109

[B11] Lawrence JM Contreras R Chen W Sacks DA Trends in the prevalence of preexisting diabetes and gestational diabetes mellitus among a racially/ethnically diverse population of pregnant women, 1999-2005 Diabetes Care 2008 31 899 904 10.2337/dc07-2345 18223030

[B12] Ferrara A Increasing prevalence of gestational diabetes mellitus: a public health perspective Diabetes Care 2007 30 Suppl 2 S141 S146 10.2337/dc07-s206 17596462

[B13] Jovanovic L Pettitt DJ Gestational diabetes mellitus JAMA 2001 286 2516 2518 10.1001/jama.286.20.2516 11722247

[B14] Dempsey JC Butler CL Sorensen TK Lee IM Thompson ML Miller RS Frederick IO Williams MA A case-control study of maternal recreational physical activity and risk of gestational diabetes mellitus Diabetes Res Clin Pract 2004 66 203 215 10.1016/j.diabres.2004.03.010 15533588

[B15] Dempsey JC Sorensen TK Williams MA Lee IM Miller RS Dashow EE Luthy DA Prospective study of gestational diabetes mellitus risk in relation to maternal recreational physical activity before and during pregnancy Am J Epidemiol 2004 159 663 670 10.1093/aje/kwh091 15033644

[B16] Flegal KM Carroll MD Ogden CL Johnson CL Prevalence and trends in obesity among US adults, 1999-2000 JAMA 2002 288 1723 1727 10.1001/jama.288.14.1723 12365955

[B17] Kim SY Dietz PM England L Morrow B Callaghan WM Trends in pre-pregnancy obesity in nine states, 1993-2003 Obesity (Silver Spring) 2007 15 986 993 10.1038/oby.2007.621 17426334

[B18] Doherty DA Magann EF Francis J Morrison JC Newnham JP Pre-pregnancy body mass index and pregnancy outcomes Int J Gynaecol Obstet 2006 95 242 247 10.1016/j.ijgo.2006.06.021 17007857

[B19] Abenhaim HA Kinch RA Morin L Benjamin A Usher R Effect of prepregnancy body mass index categories on obstetrical and neonatal outcomes Arch Gynecol Obstet 2007 275 39 43 10.1007/s00404-006-0219-y 16967276

[B20] Kramer MS Platt RW Wen SW Joseph KS Allen A Abrahamowicz M Blondel B Breart G A new and improved population-based Canadian reference for birth weight for gestational age Pediatrics 2001 108 E35 10.1542/peds.108.2.e35 11483845

[B21] ACOG Committee Opinion. Number 333, May 2006 (replaces No. 174, July 1996): The Apgar score Obstet Gynecol 2006 107 1209 1212 10.1097/00006250-200605000-00051 16648434

[B22] Solomon CG Willett WC Carey VJ Rich-Edwards J Hunter DJ Colditz GA Stampfer MJ Speizer FE Spiegelman D Manson JE A prospective study of pregravid determinants of gestational diabetes mellitus JAMA 1997 278 1078 1083 10.1001/jama.278.13.1078 9315766

[B23] Oken E Ning Y Rifas-Shiman SL Radesky JS Rich-Edwards JW Gillman MW Associations of physical activity and inactivity before and during pregnancy with glucose tolerance Obstet Gynecol 2006 108 1200 1207 10.1097/01.AOG.0000241088.60745.70 17077243 PMC1894947

[B24] Radesky JS Oken E Rifas-Shiman SL Kleinman KP Rich-Edwards JW Gillman MW Diet during early pregnancy and development of gestational diabetes Paediatr Perinat Epidemiol 2008 22 47 59 10.1111/j.1365-3016.2007.00899.x 18173784 PMC2650816

[B25] Canadian Census - 1996 Community Profile http://www12.statcan.ca/english/Profil/Details/details1.cfm?SEARCH=BEGINS&ID=7689&PSGC=35&SGC=3539036&DataType=1&LANG=E&Province=35&PlaceName=london&CMA=555&CSDNAME=London&A=&TypeNameE=City

[B26] Canadian Census - 2006 Community Profile http://www12.statcan.ca/census-recensement/2006/dp-pd/prof/92-591/details/Page.cfm?Lang=E&Geo1=CSD&Code1=3539036&Geo2=PR&Code2=35&Data=Count&SearchText=london&SearchType=Begins&SearchPR=35&B1=All&Custom=

